# Prevention and management of acute toxicities from conditioning regimens during hematopoietic stem cell transplantation

**DOI:** 10.46989/001c.94952

**Published:** 2024-04-03

**Authors:** Jana Sawyer, Taylor Elliott, Lindsay Orton, Hunter Sowell, Katie Gatwood, Kendall Shultes

**Affiliations:** 1 Pharmacy VA Tennessee Valley Healthcare System; 2 Pharmacy Vanderbilt University Medical Center

**Keywords:** Chemotherapy-induced nausea vomiting, mucositis, transplant-associated thrombotic microangiopathy, sinusoidal obstruction syndrome, conditioning toxicity

## Abstract

Hematopoietic stem cell transplantation (HSCT) remains the only curative option for several hematological malignancies. Its use has continued to grow, with an estimated 23,500 transplants performed annually in the United States alone. The acute toxicities that occur from conditioning chemotherapy can impact the peri-transplant period and have substantial implications on patients’ tolerability and outcomes, irrespective of the treatment of their disease. Chemotherapy-induced nausea vomiting (CINV), mucositis, transplant-associated thrombotic microangiopathy (TA-TMA), and sinusoidal obstruction syndrome, also known as a veno-occlusive disease (SOS/VOD) can all have significant implications for patients. These acute complications begin with the start of conditioning chemotherapy and add to potential toxicity for patients throughout the early post-transplant period, from Day +30 for CINV, mucositis, and SOS, and which can continue through at least Day +100 with the onset of TA-TMA. These toxicities must be prevented and managed appropriately. This review will summarize the literature surrounding them and guide their management.

The use of hematopoietic stem cell transplantation (HSCT), either an allogeneic HSCT (allo-HSCT) or autologous HSCT (auto-HSCT), has remained a standard of care as a curative option for patients with various hematologic malignancies. As such, the reported incidence has continued to grow across the United States, and has led to an improvement in outcomes for this patient population.^1^ With the continued rise of annual transplants, the importance of managing peri-transplantation complications remains of the upmost importance. This period typically consists of the time immediately prior to transplant during the conditioning chemotherapy, through either Day +30 or Day +100 (auto-HSCT or allo-HSCT, respectively). This review will focus on the commonly managed acute peri-transplant complications, including chemotherapy-induced nausea vomiting (CINV), mucositis, transplant-associated thrombotic microangiopathy (TA-TMA), and sinusoidal obstruction syndrome (SOS), also known as veno-occlusive disease (VOD, SOS/VOD).

## Chemotherapy Induced Nausea Vomiting

CINV is a common peri-transplant toxicity that occurs upon receipt of conditioning chemotherapy prior to HSCT. CINV can be subcategorized as acute (less than or equal to 24 hours after receipt of chemotherapy) or delayed (greater than 24 hours to 5 days after receipt of chemotherapy).^2^ CINV can also be considered anticipatory (as part of a conditioning response), radiation-induced, and/or breakthrough. It is estimated that CINV affects up to 80% of patients receiving treatment for a malignancy, and the emetogenic risk varies greatly, depending on the chemotherapeutic agent(s) received (see Table 1).^3^ Adequate prevention and management are essential to maximize the patient’s quality of life and the likelihood of maintaining enteral nutrition and adequate nutritional status.

**Table 1.** Emetic Risk Based on Chemotherapy Agents used in HSCT per NCCN Guidelines^4^

**Risk**	**Agent and Dose**
High (>90%)	Cyclophosphamide > 1,500mg/m^2^ Carmustine > 250mg/m^2^ Melphalan ≥ 140mg/m^2^ Carboplatin AUC ≥ 4 Total body irradiation (TBI)
Moderate (>30-90%)	Cyclophosphamide ≤ 1,500mg/m^2^ Carmustine ≤ 250mg/m^2^ Melphalan < 140mg/m^2^ Busulfan Methotrexate ≥ 250mg/m^2^ Cytarabine > 200mg/m^2^ Carboplatin AUC <4
Low (10-30%)	Etoposide Methotrexate > 50mg/m^2^ to < 250mg/m^2^ Cytarabine 100mg/m^2^ to 200mg/m^2^ Thiotepa
Minimal (<10%)	Fludarabine Rituximab Methotrexate ≤ 50mg/m^2^ Cytarabine < 100mg/m^2^

NCCN = National Comprehensive Cancer Institute

In addition to the type of chemotherapy agent(s) received, patient-specific risk factors for CINV can include: emesis with prior chemotherapy, female gender, prior episodes of morning sickness during pregnancy, history of motion sickness, younger age, lack of significant history of alcohol consumption or smoking, anxiety, and certain pharmacogenomic polymorphisms.^4–6^ The pathophysiology of CINV is complex and involves numerous pathways and neurotransmitters in the central and peripheral nervous systems, as well as the gastrointestinal tract.^2^ Recent developments over the past decades have elucidated targets for prevention and management.

## Pharmacologic Prevention and Management

Multiple cancer societies have developed and updated guidelines for prevention and management of CINV, including the American Society of Clinical Oncology (ASCO), the European Society for Medical Oncology (ESMO), the Multinational Association of Supportive Care in Cancer (MASCC), and the National Comprehensive Cancer Network (NCCN). Unfortunately, there is no specific guideline for HSCT patients that addresses risk based on the type of conditioning regimen. Therefore, an individualized approach should be taken based on clinical experience, patient-specific risk factors, adverse effect profile, and drug-drug and/or drug-disease state interactions. See Appendix 1 for a summary of known metabolic effects of medications commonly used in HSCT and CINV. Additionally, attention to a patient’s QT interval should be considered when selecting treatment regimens, due to cumulative QT-prolonging effects across many of the various agents.

This individualized approach should consider the use of medications across multiple drug classes, including 5-hydroxytryptamine receptor antagonists (5-HT3RAs), neurokinin 1 receptor antagonists (NK1RAs), glucocorticoids, and atypical antipsychotics (specifically olanzapine). Medications from these classes are available in a variety of dosage forms (oral, intravenous, sublingual). A patient’s clinical status should be considered when selecting the form of administration. Agents from these four drug classes should be used in combination, according to the aforementioned guidelines, as prophylaxis for patients undergoing treatment with highly or moderately emetogenic regimens. Single-agent prophylaxis may be appropriate for those treated with a regimen of low emetic risk, and no routine prophylaxis is recommended for those treated with minimally emetogenic regimens.^4^ Other agents, such as benzodiazepines and acid blockers, among others, are occasionally used, albeit less often or in specific circumstances such as for the treatment of anticipatory nausea and reflux-associated nausea, respectively.

### *5-HT3RAs* (ondansetron, granisetron, dolasetron, and palonosetron)

5-HT3RAs block serotonin peripherally on the vagal nerve terminals and centrally in the chemoreceptor trigger zone. As a class, they are generally most effective at preventing acute CINV, but palonosetron (a second-generation agent) also has efficacy in preventing delayed CINV.^7^

This class of agents is generally well tolerated, but patients should be monitored for adverse effects such as headache, constipation, elevated liver enzymes, and QT interval prolongation. Additionally, the choice of a specific agent is often institution-specific, with use of agents other than ondansetron often prohibited by cost.^2^

5-HT3RAs are primarily metabolized via the cytochrome P-450 (CYP) system of enzymes. Individuals identified as CYP2D6 ultrarapid metabolizers likely have increased metabolism of its substrates, resulting in decreased clinical benefit. In this group of patients, it is recommended by the Clinical Pharmacogenetics Implementation Consortium (CPIC) to avoid initiation of ondansetron, a minor CYP2D6 substrate, and to, instead, start an antiemetic that is not primarily metabolized by CYP2D6, such as granisetron.^8^ Although dolasetron and palonosetron are also minor substrates of CYP2D6, there is limited evidence to direct clinical decision-making for these medications in patients with genetic alterations in CYP2D6.

### *NK1RAs* (aprepitant, fosaprepitant, rolapitant, and netupitant)

NK1RAs prevent activation of the vomiting centers both centrally and peripherally by blocking the action of substance P, an excitatory motor neurotransmitter and member of the neurokinin family of neuropeptides. These medications are effective in preventing delayed nausea, as well as treating acute nausea.

The NK1RAs are also generally well tolerated, but may give rise to fatigue and dizziness. Additionally, aprepitant has been associated with increases in liver function tests.

Netupitant is only commercially available in combination with palonosetron. Several phase III clinical trials have described efficacy of netupitant + palonosetron, in combination with dexamethasone, in the prevention of both acute and delayed CINV following receipt of moderately or highly emetogenic chemotherapy regimens.^9,10^

### Glucocorticoids

The antiemetic mechanism of glucocorticoids is not well understood, although it has been proposed to be related to its anti-inflammatory action. When utilized for short periods of time, glucocorticoids may contribute to insomnia, excitation, and hyperglycemia, but are otherwise well tolerated. Prolonged use, however, can be associated with increased infection risk, which is an important consideration in the HSCT population. Their use should be avoided in patients undergoing haploidentical HSCT due to immunosuppressive effects.

### *Atypical Antipsychotics* (olanzapine)

Olanzapine is an atypical (second generation) antipsychotic. Its antiemetic effect is thought to be attributed to its central and peripheral antagonism of dopamine and serotonin receptors. Although effective as an antiemetic in acute, delayed, and breakthrough CINV, its dose-dependent anticholinergic effects can be prohibitory. Recent studies have suggested that, when compared to a 10mg dose, a 5mg dose of olanzapine resulted in similar control of CINV and oftentimes was associated with less sedation.^11–14^

### Miscellaneous

Although not used routinely for prophylaxis, the following medications may be used for treatment of breakthrough CINV or other related gastrointestinal symptoms: prochlorperazine (typical antipsychotic); promethazine (H1 antagonist); metoclopramide (dopamine/serotonin antagonist); famotidine (H2 antagonist); omeprazole, pantoprazole, lansoprazole, dexlansoprazole, esomeprazole, and rabeprazole (proton pump inhibitors); lorazepam (benzodiazepine); dronabinol (cannabinoid); calcium carbonate (antacid); simethicone (antiflatulent).

## Non-Pharmacologic Prevention and Management

In addition to appropriate pharmacologic prevention and management, there are also several non-pharmacologic strategies to prevent and manage CINV. Some of these include eating smaller, more frequent meals, eating bland foods, and eating food cold or at room temperature to decrease associated odors.^4,15^ Integrating registered dieticians into the interdisciplinary medical team can help reinforce these strategies, as well as ensure adequate nutrition goals are targeted. Other complementary and whole health-focused medical treatments include acupuncture, hypnosis, muscle relaxation, and music therapy.^4,16^

## Summary

In summary, CINV is a peri-transplant toxicity that can negatively affect patients’ quality of life and ability to maintain enteral nutrition if not adequately prevented and managed. Prevention and management should be personalized, taking into account the type of chemotherapy agent(s) used during conditioning, patient-specific risk factors, adverse effects, and potential drug-drug and drug-disease state interactions. Pharmacological strategies have evolved over the past several decades, with combination treatment from multiple antiemetic drug classes recommended for highly or moderately emetogenic regimens. Additional medications are recommended for use as breakthrough or to control other gastrointestinal symptoms that may exacerbate CINV. Non-pharmacologic prevention and management is also key to providing comprehensive care.

## Mucositis

Due to the high doses of chemotherapy, oral mucositis is one of the most common early complications of HSCT. It typically presents within the first week following conditioning therapy and lasts one to two weeks.^17^ Oral mucositis occurs as a result of deoxyribonucleic acid (DNA) damage to the epithelial cells of the oral mucosa secondary to chemotherapy and radiotherapy. Mild mucositis consists of erythematous, atrophic lesions on an intact mucosa causing a similar sensation to a food burn. Severe cases of mucositis cause ulcerations that can penetrate far within the submucosa resulting in severe pain, often necessitating narcotic analgesia and parenteral nutrition.^18^

Oral mucositis has been reported to be one of the most debilitating toxicities following HSCT with significant impacts on health, quality of life, and economic outcomes.^18^ Myeloablative HSCT recipients are among those with the highest risk of severe oral mucositis, with incidence rates up to 75%.^19^ Melphalan, busulfan and total body irradiation (TBI) are some of the most common therapies used in conditioning regimens associated with high incidences of mucositis. Other factors that contribute its incidence and severity include graft-versus-host-disease (GVHD) prophylaxis with methotrexate, female gender, and type and amount of previous chemotherapy exposure.^17,20^ Several grading scales exist, but there is no consensus on a gold standard for diagnosing and assessing the severity of mucositis. The World Health Organization (WHO) Oral Toxicity Scale is a common grading system that accounts for ulceration symptoms and ability to eat and drink. The oral mucositis assessment scale (OMAS) is another tool that includes the size of the ulcerations and the degree of erythema in its scoring.^21^ In addition to grading the ulcerations, it is important to regularly assess patients’ pain using validated instruments.

## Prevention

Despite oral mucositis being a common and debilitating toxicity, there is no standard approach to prevention and treatment.

## Good oral hygiene

Dental consults should be considered for all patients prior to HSCT to assess their oral health and need for any procedures (i.e. tooth extraction, filling). Patients should be educated to perform oral rinses 4-6 times per day using non-medicated solution such as sterile water, normal saline or sodium bicarbonate, and to brush their teeth 2-3 times per day using a soft toothbrush. Additionally, patients should be instructed to avoid alcohol-based mouth rinses.^22^

## Oral cryotherapy

Cryotherapy works by vasoconstricting the blood vessels in the oral mucosa, thereby reducing the ability of cytotoxic drugs to reach the oral tissue and cause damage. Cryotherapy is a cost-effective strategy to prevent oral mucositis; however, its use is limited to cytotoxic agents with short half-lives, mainly melphalan. Patients should be instructed to chew on ice for a short period of time prior, during and after the melphalan infusion.^22^ While this can be uncomfortable, several small studies have shown a benefit in reducing the incidence of severe melphalan-induced oral mucositis by using cryotherapy surrounding the melphalan infusion.^23,24^

## Palifermin

Palifermin is a recombinant human keratinocyte growth factor (KGF) with tissue-protective effects of increasing the mucosal epithelium thickness. It additionally upregulates transcription factor Nrf2 in keratinocytes, resulting in increased expression of reactive oxygen species detoxifying enzymes, and stimulates anti-inflammatory cytokines.^22^ Palifermin should be dosed at 60 μg/kg per day for 3 days prior to conditioning treatment, and for 3 days post-transplantation.^22^ A double-blind, phase III, multicenter study of autologous HSCT patients found that palifermin significantly reduced the incidence and duration of grade 3 or 4 mucositis and the use of opioid analgesics.^25^ Unfortunately, a major limitation to palifermin’s use in clinical practice is its associated high cost.

## Low-level laser therapy

Low-level laser therapy, now formally known as intraoral photobiomodulation (PBM) is recommended for the prevention of oral mucositis in adults undergoing HSCT with high-dose chemotherapy with or without TBI. Several dosing strategies and durations have been studied, such as administering for 5 days starting after the last day of conditioning therapy or starting with conditioning therapy and continuing for 2 days post-HSCT.^26^ Similar to palifermin, while several randomized controlled trials have found a benefit with PBM in preventing oral mucositis, a major limitation to its use is the associated economic burden.^27^

The following modalities are not recommended for prevention of oral mucositis during HSCT due to insufficient evidence supporting a benefit.^26^:

- Granulocyte-macrophage-colony stimulating factor (GM-CSF) mouthwash
- Glutamine
- Pentoxifylline
- Chlorhexidine mouthwash
- Pilocarpine

## Management

Treatment of oral mucositis is centered around supportive care for symptom relief, since mucositis is self-limiting and will improve upon engraftment.

## Pain Control

Pain relief is a vital component of oral mucositis management. It not only improves quality of life, but also can allow for resumption of oral intake and reduce hospital length of stay.

Topical therapies such as 2% viscous lidocaine mouthwash may be a helpful coating agent as an adjunct to analgesics for pain control. Topical morphine is recommended for mucositis secondary to head and neck cancer, but is not recommended for HSCT patients.^26^

Opioids are the mainstay of analgesic therapy for oral mucositis with morphine as the recommended first-line therapy.^26^ To help optimize pain relief, morphine or hydromorphone can be administered via patient-controlled analgesia (PCA) pumps.

## Total parenteral nutrition (TPN)

TPN should be considered in HSCT patients with oral mucositis who are malnourished and anticipated to be unable to ingest and absorb nutrients for a prolonged period, which is typically defined as 7-14 days. TPN should be discontinued as soon as mucositis has resolved, and the patient has adequate oral intake.^28^

## Summary

With a lack of consensus guidelines for prevention and treatment of mucositis, supportive care measures should be initiated promptly and enforced for patients throughout HSCT to avoid debilitating effects. Prevention with good oral hygiene and management of mucositis with pain control should be utilized to aid in this acute toxicity.

## Transplant-Associated Thrombotic Microangiopathy (TA-TMA)

Another acute peri-transplantation complication worth noting is TA-TMA. TMA is a condition consisting of damage to the endothelial system leading to endothelial activation and thrombosis of arterioles and venules.^29,30^ Due to microvascular thrombosis, complications such as thrombocytopenia, ischemic damage to organs, and microvascular hemolytic anemia can occur.^29^ Typically, TMA can be seen in the setting of thrombotic thrombocytopenic purpura (TTP) and forms of hemolytic uremic syndrome (HUS) as in Shiga toxin-producing *Escherichia coli*-associated (STEC-HUS) or atypical HUS (aHUS).^30^ Growing evidence exists for various causes of TMA, including complement dysregulation occurring during the transplantation process, making the need for understanding TA-TMA crucial.

Various factors throughout transplant can lead to endothelial and complement activation which can stimulate the cascade of events resulting in TA-TMA.^29^ A “three hit” hypothesis has been elucidated^29,30^:

- Patients have an underlying or pre-existing genetic predisposition or endothelial injury
- Endothelial damage occurs during the conditioning regimen
- Additional endothelial damaging injury caused by medications, alloreactivity, and/or infections

During this three-hit approach, complement becomes activated leading to microvascular thromboses and, subsequently, end organ damage. The median onset to TA-TMA varies in the post-transplant setting, but has been reported with a median interval of 19-210 days.^31,32^ Some cases of TA-TMA can be mild and self-limiting, while others can be associated with severe morbidity and mortality, with mortality rates reported as high as 75% three months after diagnosis, making it a necessity to identify and treat early in the transplant course.^31^ Delays in the diagnosis of TA-TMA have occurred due to historical limitations in consensus of diagnostic criteria. Numerous proposed definitions for the diagnosis of TA-TMA have been utilized throughout the years, leading to a variable incidence of TA-TMA reported in the literature, between 0.5%-64%, and is dependent on study population (adults versus pediatric).^30–36^ A retrospective analysis to quantify the incidence of TA-TMA from the Center for International Blood and Marrow Transplant Research (CIBMTR) database included 23,665 allo-HSCT patients and found the incidence of TA-TMA to be 2% 1-year post-HSCT and 3% at 2- and 3-years post-HSCT.^34^ Unfortunately, despite the size of the CIBMTR database, due to differences in diagnostic criteria and reporting, this number may still be underestimating the impact of TA-TMA.

Identification of patients who are most at risk for TA-TMA has been difficult in the setting of historically heterogenous diagnostic criteria. Various studies have assessed risk factors associated with TA-TMA including pre-transplant, transplant-associated, and post-transplant risk factors (Table 2).^29,30,33–36^ Calcineurin inhibitor (CNI) use alone has been difficult to identify as a risk factor, due to the majority of patients who are maintained on a CNI-based regimen.^34^

**Table 2.** TA-TMA Risk Factors^29,30,33–36^

Pre-Transplant	Transplant-Associated	Post-Transplant
Pre-existing endothelial damage/injury	Conditioning regimen type (myeloablative)	Infections: aspergillosis, cytomegalovirus, adenovirus, parvovirus B19, human herpes virus-6, and BK virus
Pre-existing kidney dysfunction	ABO incompatibility	Acute GVHD
Genetic predisposition	HLA mismatch donor (mismatch or unrelated)	GVHD regimen - use of CNI and mammalian target of rapamycin inhibitors (mTOR)
Black race		
Gender: females > males		
Primary immunodeficiency diagnosis (children)		

More recently, representatives from the American Society for Transplantation and Cellular Therapy (ASTCT), CIBMTR, Asia-Pacific Blood and Marrow Transplantation, and European Society for Blood and Marrow Transplantation (EMBT) formed an expert panel and created diagnostic and prognostic criteria for TA-TMA.^32^ This harmonized international guideline reviewed many of the commonly utilized diagnostic criteria and felt that although biopsy-proven TA-TMA is the most accurate, in the setting of HSCT, it is not always feasible. Therefore, the group felt that the modified Jodele criteria allowed for clinical and practical diagnosis with an emphasis on prevention and screening (Table 3).^32,33^ It should be noted that the majority of data surrounding the diagnosis of TA-TMA comes from the pediatric and young adult populations. Future studies are necessary in the adult HSCT population to continue to evaluate this significant complication.

**Table 3.** Diagnostic Criteria of TA-TMA^32,33^

**Biopsy-proven disease from kidney or GI tract OR**	
**Clinical criteria - must meet > 4 of 7 of the following criteria within 14 days at 2 consecutive time points**	
**Anemia**	New transfusion requirement, failure to achieve transfusion independence despite neutrophil engraftment, or hemoglobin decline of 1 g/dL from baseline
**Thrombocytopenia**	Failure to achieve platelet engraftment, higher than expected platelet transfusion needs, refractory to platelet transfusions, or 50% or greater reduction in baseline platelet count after engraftment
**Elevated LDH**	>ULN for age
**Schistocytes**	Present
**Hypertension**	>99th percentile for age (<18 years) or systolic BP >140 mmHg or diastolic BP >90mmg (>18 years)
**Elevated sC5b-9**	>ULN
**Proteinuria**	>1 mg/mg rUPCR

ULN: upper limit of normal; BP: blood pressure; rUPCR: random urine protein to creatinine ratio

## Prevention/Screening

Utilizing the newly published guidance from Schoettler et al various factors can be assessed (Table 4).^32^ This is recommended for all allo-HSCT and pediatric auto-HSCT patients with a neuroblastoma diagnosis through Day +100 post-HSCT. Screening beyond Day +100 should continue for those who have a risk factor for TA-TMA (acute Grade II-IV GVHD, extensive chronic GVHD, or infectious complications).^32^ ADAMTS13 deficiency, although commonly associated with TTP, is not typically seen in patients with TA-TMA.^29,37^ In patients who screen positive for TA-TMA, it is reasonable to assess a patient’s ADAMTS13 activity. Based on additional testing performed for when patients screen positive for TA-TMA or show clinical manifestations, high risk TA-TMA is diagnosed if any of the following are found: sC5b9 >ULN, rUPCR > 1mg/mg, organ dysfunction, lactate dehydrogenase (LDH) >2X ULN, concurrent grade 2-4 acute GVHD, or concurrent viral infections.^32^

**Table 4.** TA-TMA Screening^32^

**Day 0 through discharge or Day +30**	**Day +30 to Day +100 or beyond if risk factors present**
Daily: complete blood counts, chemistry, BP Twice weekly: LDH Weekly: schistocytes, rUPCR	Weekly: complete blood counts, chemistry, LDH, schistocytes, BP, rUPCR
**A positive screen for TA-TMA is any 3 of 6 features: anemia, thrombocytopenia, elevated LDH, schistocytes, refractory hypertension, or proteinuria**	
**Patients with a positive screen for TA-TMA or clinical concern, should have the following additional tests performed:** sC5b-9, manual review of peripheral blood smear, ADAMTS13 activity, Coombs (direct and indirect), Disseminated Intravascular Coagulation (DIC) panel, haptoglobin, plasma free hemoglobin, additional organ function testing as indicated	

Multiple organ systems can be involved in TA-TMA and should be assessed for potential disease. Typical areas for TA-TMA include the CNS, with signs and symptoms being seizures, altered mental status, posterior reversible encephalopathy syndrome (PRES) and renal dysfunction. The latter and the development of proteinuria, hypertension or renal failure are associated with poor prognosis.^30^ Additional areas of involvement include gastro-intestinal tract presenting with abdominal pain, ischemia, and bleeding; cardio-pulmonary- including pulmonary hypertension; serositis, diffuse alveolar hemorrhage, and hypertension. Close monitoring is pertinent, as hypertension (often requiring > 1 antihypertensive agent) can be an early sign of TA-TMA.^33^

## Management

Numerous studies have explored potential treatment options for TA-TMA which include targeted treatment as well as management of risk factors.^31,38^ In the more recently published harmonized international guidelines, TA-TMA management is recommended according to diagnosis of either standard risk or high-risk TA-TMA. The latter patients should be considered for targeted therapy and management of concurrent complications, whereas standard risk patients should have risk factors aggressively managed but continue with vigilant monitoring.^32^

## Therapeutic Plasma Exchange (TPE)

TPE is typically useful in TTP as it will remove the anti-ADAMTS13 antibodies. Caplacizumab, an anti-von Willebrand Factor monoclonal antibody has Food and Drug Administration (FDA) approval for acquired-TTP and is used with TPE.^39^ Unlike in TTP, plasma exchange data show limited efficacy in TA-TMA, most likely due to the majority of patients with TA-TMA having normal (>5%) ADAMTS13 levels. There are no studies evaluating the use of caplacizumab for TA-TMA.^30,36,37^ Data surrounding TPE are so limited that in 2005, the toxicity committee of the Blood and Marrow Transplant Clinical Trials Network (BMT-CTN), recommended it not be utilized without definitive trials.^40^

## Rituximab

The exact mechanism for use of rituximab in TA-TMA is unknown. Current evidence is limited and supported by case reports, when it is typically used in patients with TTP in combination with TPE or for those unresponsive to TPE.^41^ Au et al reported the use of weekly rituximab 375mg/m^2^ x 4 in 5 patients who were refractory to TPE. Four had a positive hematological response.^42^ Jodele et al reported success with rituximab when given concurrently with TPE as an early intervention in a pediatric patient.^43^

## Reduction in immunosuppression or discontinuation of CNI

The immediate reduction or discontinuation of a CNI has to be clinically balanced with the concern for precipitating or worsening GVHD, another known risk factor for TA-TMA.^40,44^

Ho et al and the BMT-CTN toxicity committee recommended discontinuation of CNI after the diagnosis of TA-TMA.^40^ A retrospective study of 1219 allo-HSCT patients found that the use of tacrolimus, regardless of supratherapeutic or therapeutic levels at the time of diagnosis, had similar outcomes. Additionally, if patients had tacrolimus discontinued, their outcomes were similar to those who were maintained on it but at a lower level.^45^

In a single institution study, complete withdrawal of CNI had no effect on hematological outcomes or mortality, compared to continuation of the CNI. The combination of sirolimus (with higher troughs) and CNIs was found to be an independent risk factor for TA-TMA development.^46^

Conversely, in the largest study in TA-TMA patients, Matsui et al retrospectively reviewed 2425 adult allo-HSCT patients and found an incidence of 5% of TA-TMA at a median of 35 days post-HSCT. Modulation of CNI use after diagnosis was divided into patients who were maintained on CNI or reduced, switched to another CNI, switched to corticosteroids, or increased corticosteroid dose without adjusting CNI. Patients who were continued on CNI or had doses slightly reduced were found to have improved response rates as well as improved overall survival (OS), compared to those who were switched to corticosteroids (median OS: 50 days; 95% CI, 36-75 versus 17 days; 95% CI, 7-41, P<0.01).^47^

## Defibrotide

Although not FDA approved for TA-TMA, due to its endothelial protective effects, some case reports and a prospective pilot study have utilized defibrotide in TA-TMA.^48^ Defibrotide is administered as a weight-based intravenous infusion every 6 hours, but no set dose is provided for treatment of TA-TMA.

Twelve patients were given defibrotide an average of 2 days after TA-TMA diagnosis, with 9 having varying degrees of response. Of note, six deaths occurred due to unrelated complications including infections, GVHD and disease progression.^49^

In another case series, 11 of 17 (65%) adult allo-HSCT patients who received defibrotide either alone or in combination with other therapies (rituximab or TPE) achieved complete response. Bleeding events were reported in 4 (23%) patients.^50^

In a pilot study of 25 patients who met high-risk criteria for TA-TMA, the use of prophylactic defibrotide from the start of conditioning through Day +21 post-transplant resulted in a low 4% incidence of TA-TMA, compared to historical values of 18-40%. Clinically significant bleeding events led to discontinuation of defibrotide in 12% of patients.^51^

Yaetes et al retrospectively reviewed 39 patients (22 pediatrics, 17 adults) undergoing allo-HSCT with TA-TMA and treatment with defibrotide initiated at a median time of 0 days (pediatric group) or 1 day (adult group) of TA-TMA diagnosis. Resolution of TA-TMA occurred in 77% of both pediatric and adult patients, and the mortality was found to be significantly lower than in the unresolved TA-TMA group (p=0.006). Resolution of TA-TMA was higher in patients who were diagnosed and received earlier intervention. It should be noted that the doses used were higher than FDA approved dosing.^52^

## Eculizumab

Given the role that complement activation has in the development of TA-TMA, many studies have evaluated the use of eculizumab, which is currently FDA approved for aHUS.^53^ Although other complement inhibitors, ravulizumab and nomacopan, are currently under investigation for the treatment of TA-TMA, this article will focus on currently available literature surrounding eculizumab in TA-TMA.^54^ Eculizumab is a monoclonal antibody to C5 that binds and inhibits the cleavage of C5a and C5b, preventing the formation of the C5b-9terminal complex, which has been found to be elevated in patients with TA-TMA.^33,53^

Most data on the use of eculizumab come from the pediatric population. Jodele et al showed responses in 4 of 6 pediatric patients who had achieved therapeutic eculizumab levels of >99μg/mL and had complete renal recovery.^55^ It should be noted that patients required higher than FDA-approved dosing in this study.

In a case series of 5 adult patients who did not show improvement after 4 weeks of tacrolimus discontinuation and were treated with the FDA-approved dosing of eculizumab 4 responded to this drug, achieved transfusion independence, and had renal recovery.^56^

Longer-term results seem to vary. In a retrospective review of 12 patients, (3 children and 9 adults) eculizumab led to about 50% hematological response and 33% OS at the 14-month follow-up.^57^ Dhakal et al reviewed 26 cases of TA-TMA and the use of eculizumab, with 9 being post-HSCT. They found a 78% recovery rate, compared with 100% in solid organ transplant, at a median follow-up of 52 weeks, with no reported additional deaths.^58^

In a large retrospective study in 64 pediatric patients with high-risk TA-TMA, responses continued to show the efficacy of eculizumab with a 66% survival rate one year post-HSCT. Eculizumab was able to be stopped due to resolution of TA-TMA after a median of 66 days.^59^

## Supportive care

As there is a lack of robust standard treatments for TA-TMA, these additional supportive care measures are also recommended and utilized in combination with any of the treatments addressed within this review.

- Management or early identification of bacterial or viral infections
- Management of underlying GVHD diagnosis
- Management of hypertension (including avoiding nephrotoxic agents)

## Summary

TA-TMA is a complicated and life-threatening condition for patients undergoing HSCT. Early recognition and management are essential for adjustment in care to improve patients’ outcomes. Based on recent harmonization international guidelines, it is recommended for thorough screening to occur through Day +100 or longer, based on patient specific risk factors. In those who meet high-risk criteria for TA-TMA, use of targeted therapies such as eculizumab is recommended.

## Sinusoidal Obstruction Syndrome (SOS)/Hepatic Veno-Occlusive Disease (VOD)

VOD/SOS is a potentially life-threatening complication associated with HSCT.^60,61^ The mean overall incidence is estimated to be between 8% to 14% following HSCT; however, individual studies have reported highly variable rates, ranging from 0% to 62%.^62–64^ These discrepancies are due to variability in clinical diagnostic criteria, presence of differential diagnoses that are similar to VOD/SOS, and differences in patient risk factors for developing VOD/SOS.^65^ HSCT outcomes are significantly affected by VOD/SOS, and it has been reported as a leading cause of death in the early post-HSCT period. The associated mortality rate is 80% in patients who develop VOD/SOS with concomitant multiorgan dysfunction.^63,66^

VOD/SOS is thought to arise from sinusoidal endothelial cell damage secondary to high-dose conditioning chemotherapy and/or irradiation that activates proinflammatory pathways, coagulation cascades, and fibrinolytic pathways.^61,64,65^ The pathophysiologic cascade is characterized by loss of thrombolytic-fibrinolytic balance, endothelial dissection, sinusoidal lumen reduction, and occlusion of the microcirculation.^61,67–69^ This can eventually lead to hepatorenal syndrome and other multiorgan dysfunction.^60,61,67,69^

Awareness of risk factors for developing VOD/SOS is critical in the prevention and early detection of disease. These have been previously categorized into two groups: pre-transplantation patient characteristics and transplant-related factors.^65^

Pre-transplantation patient-related factors associated with an increased risk of VOD/SOS in adults^65,67^ are baseline elevations of transaminases and bilirubin, iron overload (serum ferritin levels >950 ng/mL), prior liver disease, older age, decreased performance status (ECOG 2-4 or KPS <90%), certain underlying diseases such as relapsed or refractory acute leukemias, sickle cell disease, and myelofibrosis and, in females, the use of norethindrone.^65^

In pediatric patients, increased risk of VOD/SOS is observed for age <2 year and the presence of underlying conditions, such as hemophagocytic lymphohistiocytosis and osteopetrosis^65,67^

Another important patient-related risk factor in both adult and pediatric patients includes prior gemtuzumab ozogamicin (GO) or inotuzumab ozogamicin exposure.^70,71^ The published data on this are conflicting, as a recent retrospective analysis in patients with acute myeloid leukemia who previously received GO reported that the incidence of all VOD/SOS and severe VOD/SOS was 4% and 3%, respectively compared to 3% and 1% in patients without GO exposure.^72^

Key transplant-related factors that increase the risk of VOD/SOS in both adults and pediatrics^65,67^ are allo-HSCT versus auto-HSCT, unrelated and mismatched donors, myeloablative conditioning regimens, oral busulfan versus intravenous pharmacokinetic adjusted busulfan, second HSCT and the use of sirolimus or cyclosporine for GVHD prophylaxis. For pediatric patients, the receipt of tandem autologous HSCT for neuroblastoma also increases the risk for VOD/SOS.^65,67^

VOD/SOS can be highly variable in its clinical course and severity of symptoms. The disease is characterized by fluid overload, ascites, rapid weight gain, hepatomegaly, jaundice, and right upper quadrant pain, in the absence of other causes.^63,65^ Patients presenting with severe VOD/SOS have concomitant multi-organ dysfunction, such as respiratory, renal, or cardiac. Symptoms typically occur within 21 days of HSCT; however, there have been reports of VOD occurring later than 30 days after HSCT, even up to Day +100.^73^ In addition to clinical signs, laboratory findings that may be seen with VOD/SOS include elevated total bilirubin, serum transaminases, and serum creatinine, hypoxemia, decreased protein C, and antithrombin, and transfusion-refractory thrombocytopenia.^63,73,74^ Abdominal ultrasound with Doppler is a non-invasive and useful tool that can aid in the identification of hepatomegaly, ascites, gallbladder wall edema, and reduced or reversed hepatic venous flow, which can confirm the clinical diagnosis of VOD/SOS.^63,75^ Additionally, liver stiffness measurement (LSM) through magnetic resonance elastography has been identified as a potential surrogate for portal hypertension. In recent studies, LSM has been shown to be both sensitive and specific to VOD/SOS, at 75% and 98.7%, respectively. Increases in LSM at Day +14 allowed for early detection of VOD/SOS with specificity of 99% and, as such, this can be a useful tool in VOD/SOS diagnosis, and may eventually be incorporated into future diagnostic criteria.^76^

While VOD/SOS symptoms have been well described in the existing literature, none are specific to VOD/SOS, which often makes diagnosis challenging, particularly since it is based on retrospectively developed clinical criteria. The two validated diagnostic criteria for VOD/SOS are the Baltimore and the modified Seattle criteria, which differ in the number and magnitude of clinical criteria that are required for diagnosis.^77,78^ Commonalities between each set include total bilirubin >2 mg/dL, hepatomegaly, ascites, and weight gain. Some of the main limitations with both criteria include time constraint of VOD/SOS occurrence within 21 days following HSCT, recent use of imaging techniques that are more sensitive and specific to VOD/SOS, and more recent clinical descriptions of the disease as compared to those described 25 years ago. The EBMT developed diagnostic criteria in 2016 that allows for diagnosis beyond Day +21 if the patient satisfies the Baltimore criteria, has histologically proven VOD/SOS, or meets two of the classical clinical criteria in addition to demonstrating hemodynamic and/or ultrasound evidence of VOD/SOS.^79^ These criteria have been further updated in 2023, and now categorize VOD/SOS as probable, clinical, and proven. Proven VOD/SOS requires histological evidence of disease or documented elevated hepatic venous wedge pressure.^76^ Similarly, the Cairo/Cooke diagnostic criteria have also removed the time constraint for VOD/SOS onset, and include ultrasound evidence of VOD/SOS and transfusion-refractory thrombocytopenia as clinical criteria.^80^ While the Baltimore and Seattle criteria are the most validated diagnostic tools, the EBMT and Cairo/Cooke criteria are increasingly used in clinical practice, based on the incorporation of more comprehensive, sensitive, and specific criteria for VOD/SOS detection.

## Prevention

Prevention of VOD/SOS is of great importance because of the high mortality rate and limited treatment options. All patients undergoing HSCT should be carefully screened for risk factors of VOD/SOS development, and undergo close monitoring throughout HSCT for VOD/SOS symptoms. Prevention also includes avoidance of hepatotoxic medications and modifying transplant regimens, to reduce transplant-related risk factors where possible, as well as efforts to reduce fluid overload.^65^ Currently, there is no consensus recommendation on risk stratification of patients to determine who should receive pharmacologic prophylaxis, and no agent has approval for VOD/SOS prevention in the United States.

## Ursodeoxycholic acid (ursodiol)

Ursodiol is a naturally occurring hydrophilic bile acid that is thought to reduce the potential for liver damage by increasing the amount of hydrophilic acid in the bile acid pool. In comparison to placebo, two prospective randomized trials have shown statistically significant reductions in the incidence of VOD with ursodiol 600-900 mg by mouth daily when started at the initiation of conditioning chemotherapy.^81,82^

On another prospective randomized trial of 242 patients undergoing allo-HSCT, ursodiol 12 mg/kg/day by mouth from the day prior to conditioning chemotherapy to Day +90 after HSCT did not significantly reduce the incidence of VOD/SOS; however, the ursodiol group had reduced rates of hepatic complications, lower incidence of severe acute GVHD (4% versus 14.2%, p=0.01), and improved survival at one-year (71% versus 55%, p=0.02).^83^

Also, a systematic review including three randomized trials comparing prophylactic ursodiol to no prophylaxis demonstrated a reduction in VOD/SOS (relative risk [RR], 0.34; 95% CI, 0.17-0.66) and transplant-related mortality (RR, 0.58; 95% confidence interval [CI] 0.35-0.95).^84^

Therefore, ursodiol is generally accepted as standard of care for VOD/SOS prevention in patients undergoing allo-HSCT, based on the available data, along with its favorable toxicity profile and ease of administration.

## Defibrotide

Defibrotide is a complex of single-stranded oligodeoxyribonucleotides derived from porcine intestinal mucosal DNA thought to provide endothelial protection and fibrinolytic balance.^85^ Defibrotide is the only FDA-approved agent for treatment of VOD/SOS in adults or pediatrics, but its use for prevention of VOD/SOS is controversial.

A meta-analysis of five controlled studies showed a significant reduction in the incidence of VOD/SOS in patients at high-risk of developing VOD/SOS (RR, 0.47; 95% CI, 0.31-0.73), but there was significant heterogenicity between each of the trials included.^86^

In a phase III, randomized, controlled trial, defibrotide significantly reduced the risk of VOD/SOS when compared to the control group in patients ≤18 years-old at high-risk of developing VOD/SOS (risk difference, -7.7%; 95% CI, 15.3-20.1; p=0.0488).^87^

Conversely, when defibrotide was added to supportive care in patients with a high or very high-risk of VOD/SOS in a phase III randomized controlled trial, there was no benefit in the rate of VOD/SOS-free survival at Day +30 (hazard ratio [HR], 1.27; 95% CI 0.84-0.93).^88^

Given these data, along with its cost, toxicity risk, and intravenous route of administration, defibrotide is not routinely recommended for use as VOD/SOS prophylaxis.

## Management

Treatment of VOD/SOS is limited to aggressive supportive care and defibrotide. In 2016, defibrotide was approved for use in the United States for the treatment of VOD/SOS with renal or pulmonary dysfunction following HSCT.^48^

In the pivotal phase III trial evaluating the use of defibrotide, there was a lower Day +100 mortality rate and higher Day +100 VOD/SOS complete response rate with defibrotide when compared to historical controls.^89,90^ In additional studies, defibrotide was demonstrated to lead to complete response rates ranging from 42% to 76% and Day +100 survival rates ranging from 32% to 79%.^89–96^

The dosing of defibrotide is 6.25 mg/kg intravenously every six hours, and is recommended to be given for a minimum of 21 days up to a maximum of 60 days if VOD/SOS signs or symptoms have not resolved.^97^

The most common adverse effects are hypotension, diarrhea, vomiting, nausea, and epistaxis, and it has been associated with significant bleeding in a minority of patients. It should not be initiated in patients with active bleeding, and patients should be monitored closely for bleeding while on therapy. Concomitant use of systemic anticoagulants or fibrinolytics with defibrotide is contraindicated.

It is also critically important to initiate treatment promptly in patients meeting criteria for VOD/SOS diagnosis who have evidence of renal or pulmonary dysfunction before progression to severe disease. There have been multiple retrospective analyses that have observed significantly worse prognosis and outcomes if treatment is delayed by even as little as two days.^89,94,98^

## Summary

Given the high mortality associated with VOD/SOS, prevention, if possible, and early intervention are essential for improving outcomes. For our summary on prevention, monitoring and management, please see Appendix 2.

## Conclusion

Acute complications associated with conditioning regimens in HSCT patients are important to identify and to promptly manage. Table 5 includes a summary of all complications discussed within this review, as well as considerations based on the type of HSCT. With the continued growth of HSCT, recognition and care throughout the peri-transplantation period is imperative to avoid unnecessary toxicity and provide best outcomes for patients.

**Table 5.** Summary of acute toxicity management strategies in HSCT patients

Peri-Transplantation Complication	**Onset of Complication**	**Monitoring**	**Management**	**Considerations Based on Transplant Type**	
Chemotherapy Induced Nausea Vomiting	Acute: < 24 hours after chemotherapy Delayed: > 24 hours after chemotherapy	Frequency of emesis Control of nausea Electrolytes, fluid status Nutritional status	Prophylaxis dependent upon emetogenic risk of conditioning regimen Pharmacologic and non-pharmacologic interventions	*Allo-HSCT vs auto-HSCT* Based on emetogenic risk of chemotherapy agent(s) used; see Table 1 *Conditioning Type* Based on emetogenic risk of chemotherapy agent(s) used; see Table 1 Use of multiple agents may escalate CINV risk *Donor Source* Type of donor does not affect CINV risk *GVHD regimen* Higher risk if post-transplant cyclophosphamide used	
Mucositis	Within the 1^st^ week following conditioning chemotherapy	Physical oral exam Oral intake and nutrition status Complete blood counts Pain scores	Self-limiting Supportive care: pain relief and parenteral nutrition	*Allo-HSCT vs auto-HSCT* Similar risk depending on level of myeloablation from conditioning regimen *Conditioning Type* Myeloablative conditioning increased risk over reduced intensity conditioning Regimens containing busulfan, melphalan, etoposide or TBI increased risk *Donor Source* Type of donor does not affect mucositis risk *GVHD regimen* Methotrexate containing GVHD regimens increased risk	
Transplant-Associated Thrombotic Microangiopathy	Median interval of 19-210 days	Complete blood counts, chemistry, BP, LDH, schistocytes, rUPCR Frequency varies based on time from transplant	Standard-Risk patients: supportive care and aggressive management of risk factors High-Risk patients: targeted therapy (eculizumab) in combination with aggressive management of risk factors	*Allo-HSCT vs auto-HSCT* Higher risk in allo-HSCT and auto-HSCT pediatric patients with neuroblastoma diagnosis *Conditioning Type* Higher risk with myeloablative conditioning over reduced intensity conditioning *Donor Source* HLA mismatch donors (mismatched or unrelated) are at increased risk *GVHD regimen* CNI-based or mTOR-based regimens increased risk	
Veno-Occlusive Disease (VOD/SOS)	Median of 19 days (MAC) and 26 days (RIC)^99,100^	CMP (to assess AST, ALT, total bilirubin) at least twice weekly BMP daily CBC daily Weight and fluid balance daily Abdominal ultrasound with Doppler if LFTs become elevated	Mild cases: Supportive care including diuresis, discontinuation of hepatotoxic medications, and platelet transfusions Moderate-severe cases: Supportive care including diuresis, discontinuation of hepatotoxic medications, platelet transfusions, sodium restriction, supplemental oxygen, paracentesis, dialysis, mechanical ventilation Defibrotide 6.25 mg/kg IV q6h for at least 21 days	*Allo-HSCT vs auto-HSCT* Higher risk in allo-HSCT over auto-HSCT Higher risk with second allo-HSCT *Conditioning Type* Higher risk with myeloablative conditioning vs reduced intensity conditioning Higher risk with regimens containing oral busulfan vs intravenous pharmacokinetic adjusted busulfan *Donor Source* HLA mismatch donors (mismatched or unrelated) are at increased risk *GVHD regimen* Higher risk with sirolimus- or cyclosporine-containing regimens	

Abbreviations: MAC: myeloablative conditioning, RIC: reduced intensity conditioning; CMP: complete metabolic panel; AST: aspartate aminotransferase; ALT: alanine transaminase; BMP: basic metabolic panel; CBC: complete blood count; LFTs: liver function tests; BP: blood pressure; LDH: lactate dehydrogenase; rUPCR: random urine protein to creatinine ratio

### Competing interest

Katie Gatwood has received a speaker honorarium from Jazz Pharmaceuticals.

### Authors’ Contribution per CRediT

Conceptualization: Kendall Shultes; Methodology: Kendall Shultes, Jana Sawyer, Project Administration and supervision: Kendall Shultes, Writing – original draft: Kendall Shultes, Jana Sawyer, Taylor Elliott, Lindsay Orton, Hunter Sowell, Katie Gatwood; Writing – review & editing: Kendall Shultes, Jana Sawyer, Taylor Elliott, Lindsay Orton, Hunter Sowell, Katie Gatwood

## Appendix

**Appendix 1.** Summary of known metabolism effects of medications commonly used in HSCT and CINV

Medication	Substrate	Inducer	Inhibitor
Tacrolimus	CYP3A4 (major), P-glycoprotein/ABCB1 (major)		
Mycophenolate mofetil	OAT1/3, OATP1B1/1B3 (SLCO1B1/1B3), UGT1A10, UGT1A8, UGT1A9, UGT2B7		
Cyclosporine	CYP3A4 (major), P-glycoprotein/ABCB1 (major)		BCRP/ABCG2, BSEP/ABCB11, CYP2C9 (weak), CYP3A4 (weak), OATP1B1/1B3 (SLCO1B1/1B3), P-glycoprotein/ABCB1
Cyclophosphamide	CYP2A6 (minor), CYP2B6 (major), CYP2C19 (minor), CYP2C9 (minor), CYP3A4 (minor)		
Etoposide	CYP1A2 (minor), CYP2E1 (minor), CYP3A4 (major), P-glycoprotein/ABCB1 (major)		
Carmustine	CYP1A2 (minor), CYP2E1 (minor), CYP3A4 (major), P-glycoprotein/ABCB1 (major)		
Busulfan	None known		
Fludarabine	None known		
Rituximab	None known		
Thymoglobulin	None known		
Melphalan	None known		
Ondansetron	CYP1A2 (minor), CYP2C9 (minor), CYP2D6 (minor), CYP2E1 (minor), CYP3A4 (minor), P-glycoprotein/ABCB1 (minor)		
Palonosetron	CYP1A2 (minor), CYP2D6 (minor), CYP3A4 (minor)		
Olanzapine	CYP1A2 (major), CYP2D6 (minor), UGT1A4		
Fosaprepitant	CYP1A2 (minor), CYP2C19 (minor), CYP3A4 (major)	CYP2C9 (weak)	CYP3A4 (weak)
Promethazine	CYP2B6 (minor), CYP2D6 (minor)		
Prochlorperazine	None known		
Lorazepam	None known		

### Appendix 2. Summary VOD/SOS Recommendations for Monitoring and Management

Prevention

1. Avoid hepatotoxic drugs in patients at high-risk for VOD/SOS, if possible. Assessment of offending medications should be made daily.
2. We recommend initiating ursodiol 300 mg by mouth three times daily for VOD/SOS prophylaxis, beginning the day prior to starting conditioning chemotherapy until day +90, in all patients undergoing allo-HSCT. We also recommend considering ursodiol use in high-risk patients undergoing auto-HSCT.

Monitoring

1. All patients undergoing HSCT should be screened for risk factors of developing VOD/SOS. Identifying these prior to HSCT is an opportunity to account for the patient’s risk by altering modifiable transplant-related risk factors.
2. Basic metabolic panel and complete blood count should be checked daily in patients undergoing allo-HSCT until at least day +30 if the patient does not have suspected VOD/SOS. Complete metabolic panel should be monitored at least twice weekly, specifically addressing trends in total bilirubin, serum transaminases and serum creatinine, noting changes from the patient’s baseline. If liver function tests begin to rise, a complete metabolic panel should be obtained daily.
3. Weight and fluid balance should be monitored at least daily to assess for volume overload and need for diuresis. Monitoring frequency should increase if patients begin to develop signs of fluid overload or other early VOD/SOS symptoms.
4. In patients who have clinical symptoms of VOD/SOS, we recommend obtaining a Doppler ultrasound to assess for reversal of portal venous flow and/or rule-out other causes of liver dysfunction.

Management

1. We recommend the following aggressive supportive measures in all patients diagnosed with VOD/SOS:
  - Sodium and fluid restriction with diuresis to maintain euvolemia and adequate renal perfusion
  - Discontinuation of hepatotoxic and nephrotoxic medications if agents are not essential
  - Platelet transfusions
  - Supplemental oxygen in event of hypoxia
  - Paracentesis if significant ascites
  - Renal replacement therapy in event of renal failure
  - Mechanical ventilation in event of respiratory failure
2. In addition to aggressive supportive measures, we recommend initiation of defibrotide in patients with a confirmed diagnosis of VOD/SOS with renal or pulmonary dysfunction
  - Defibrotide should be initiated as soon as possible after VOD/SOS diagnosis, but no later than two days following diagnosis
  - Defibrotide should be dosed at 6.25 mg/kg IV every six hours for at least 21 days and up to a maximum of 60 days
  - Use baseline body weight from pre-HSCT to determine defibrotide dosing weight
3. In patients on defibrotide, we recommend the following monitoring parameters:
  - AST, ALT, bilirubin
  - INR
  - Fibrinogen
  - Defibrotide-related toxicities, such as bleeding and allergic reactions
4. In patients on defibrotide, we recommend the following supportive care measures to decrease bleeding risk:
  - Transfuse platelets to maintain platelet count >30,000/microliter
  - Transfuse packed red blood cells to maintain hematocrit > 30%
  - Administer cryoprecipitate to maintain fibrinogen >150 mg/dL
  - Administer vitamin K to maintain INR < 1.5 and prothrombin time <15 seconds
